# Supplementary material for: A distinct class of pan-cancer susceptibility genes revealed by an alternative polyadenylation transcriptome-wide association study
Source: Nat Commun. 2024 Feb 26;15:1729. doi: 10.1038/s41467-024-46064-7 (PMC10897204; doi:10.1038/s41467-024-46064-7)
Supplement: Supplementary file 1 — Supplementary Information [file 41467_2024_46064_MOESM1_ESM.pdf]

Supplementary Information for

A distinct class of pan-cancer susceptibility genes revealed by an alternative polyadenylation transcriptome-wide association study

Hui Chen, Zeyang Wang, *et al*.

*Corresponding author:

Wei Li, wei.li@uci.edu

Lin Deng, denglin@szbl.ac.cn

Lei Li, lei.li@szbl.ac.cn

The PDF file includes:

- Figures S1 to S23
- Supplementary Methods

Supplementary Figures

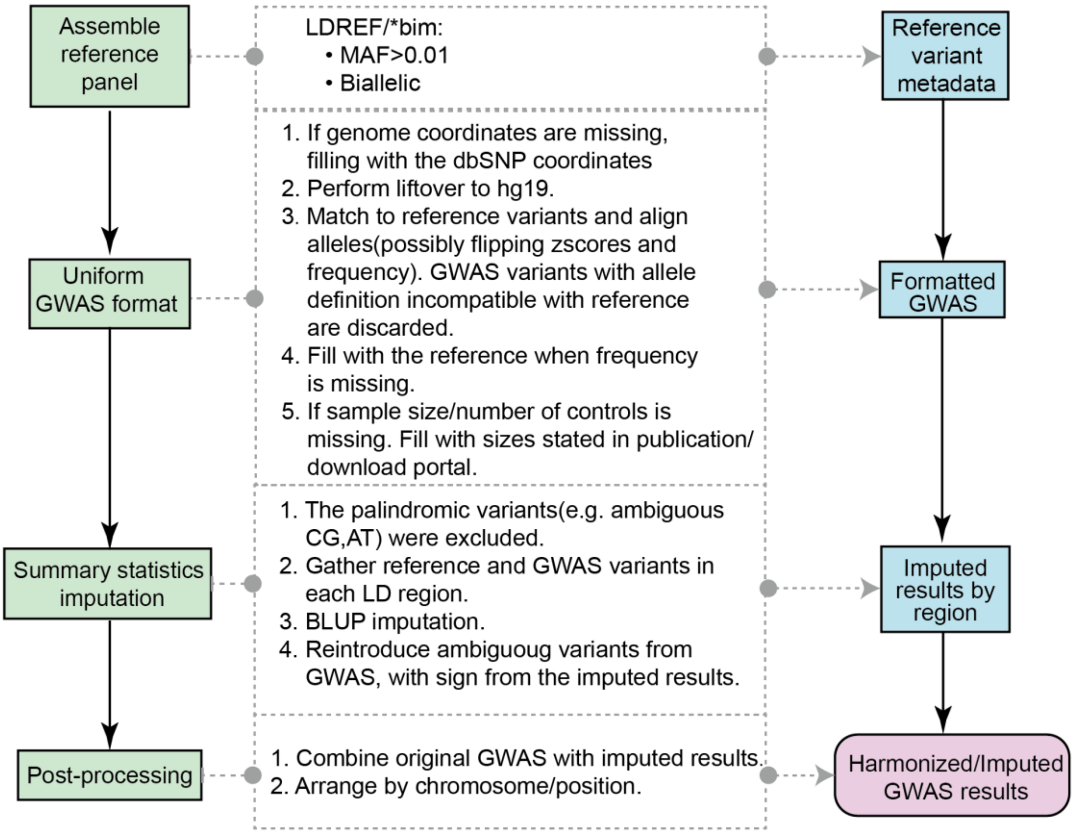

Figure S1. Workflow for cancer GWAS summary statistics analysis.

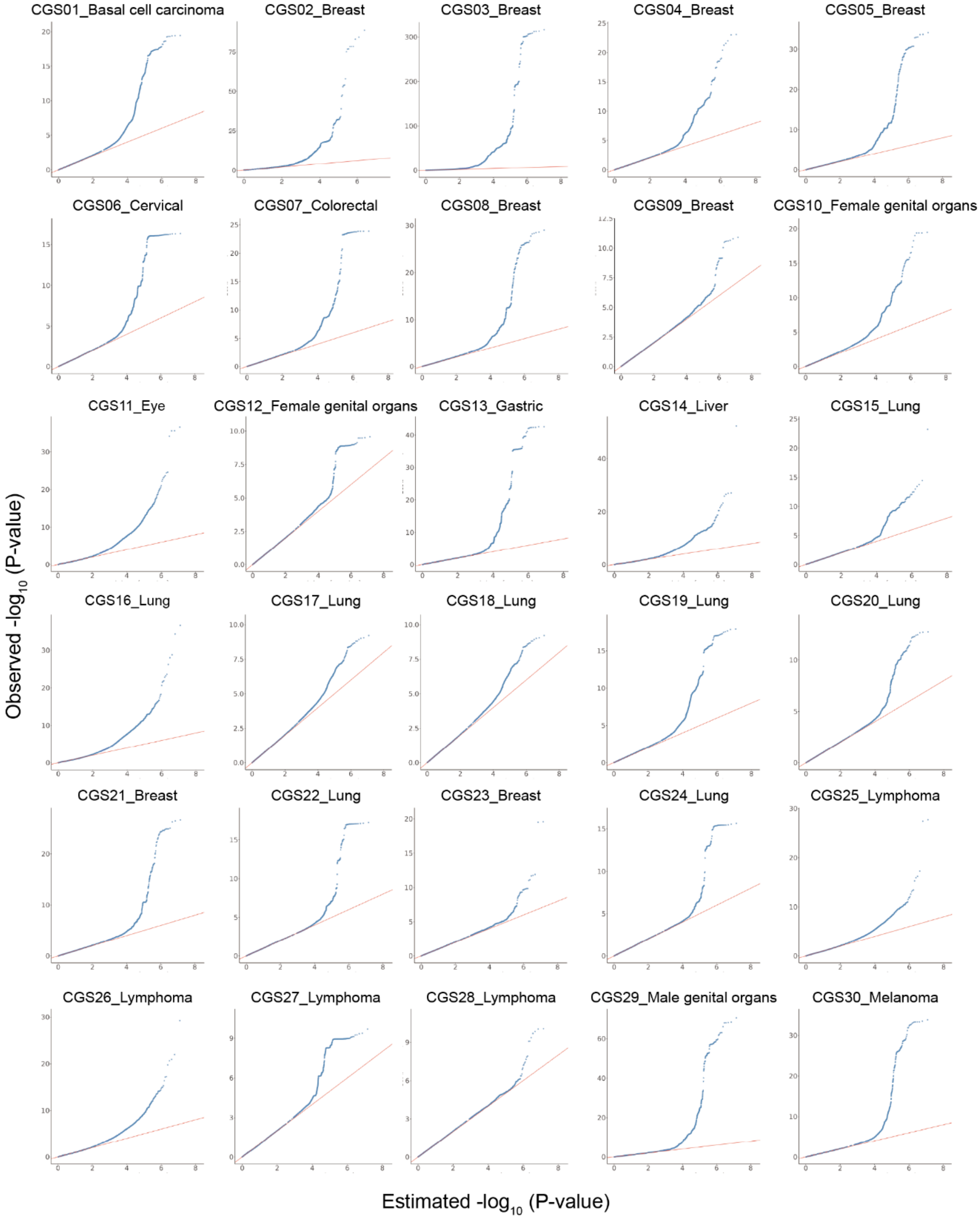
CGS01_Basal cell carcinoma
CGS02_Breast
CGS03_Breast
CGS04_Breast
CGS05_Breast
CGS06_Cervical
CGS07_Colorectal
CGS08_Breast
CGS09_Breast
CGS10_Female genital organs
CGS11_Eye
CGS12_Female genital organs
CGS13_Gastric
CGS14_Liver
CGS15_Lung
CGS16_Lung
CGS17_Lung
CGS18_Lung
CGS19_Lung
CGS20_Lung
CGS21_Breast
CGS22_Lung
CGS23_Breast
CGS24_Lung
CGS25_Lymphoma
CGS26_Lymphoma
CGS27_Lymphoma
CGS28_Lymphoma
CGS29_Male genital organs
CGS30_Melanoma
Observed -log10 (P-value)
Estimated -log10 (P-value)

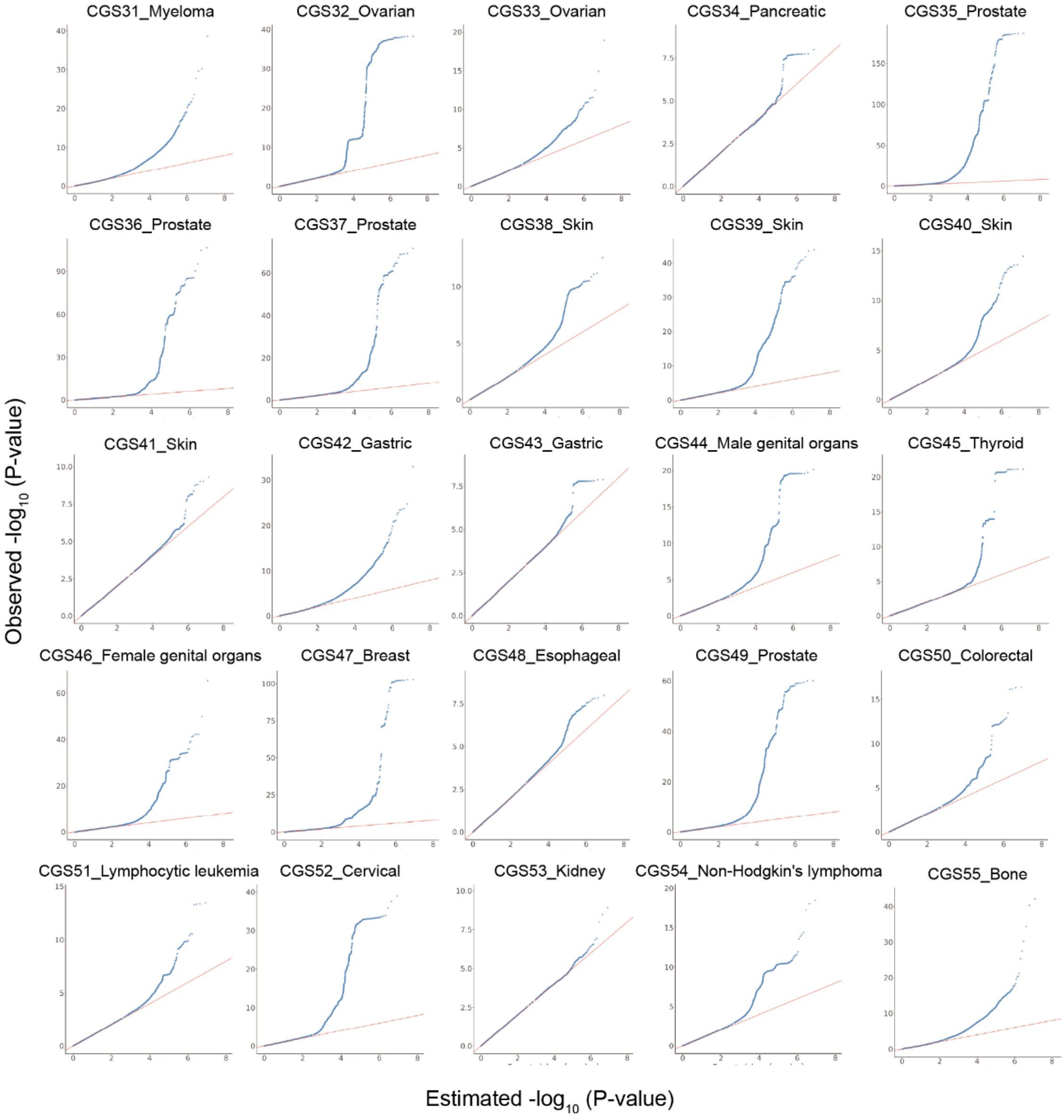

Figure S2. Quantile-Quantile (QQ)-plot of GWAS summary data. QQ-plot shows the quality control for each cancer trait, and generally plots the observed $-\log_{10}$*P*-value (y-axis) versus the quantile distribution of excepted $-\log_{10}$ *P*-value (x-axis).

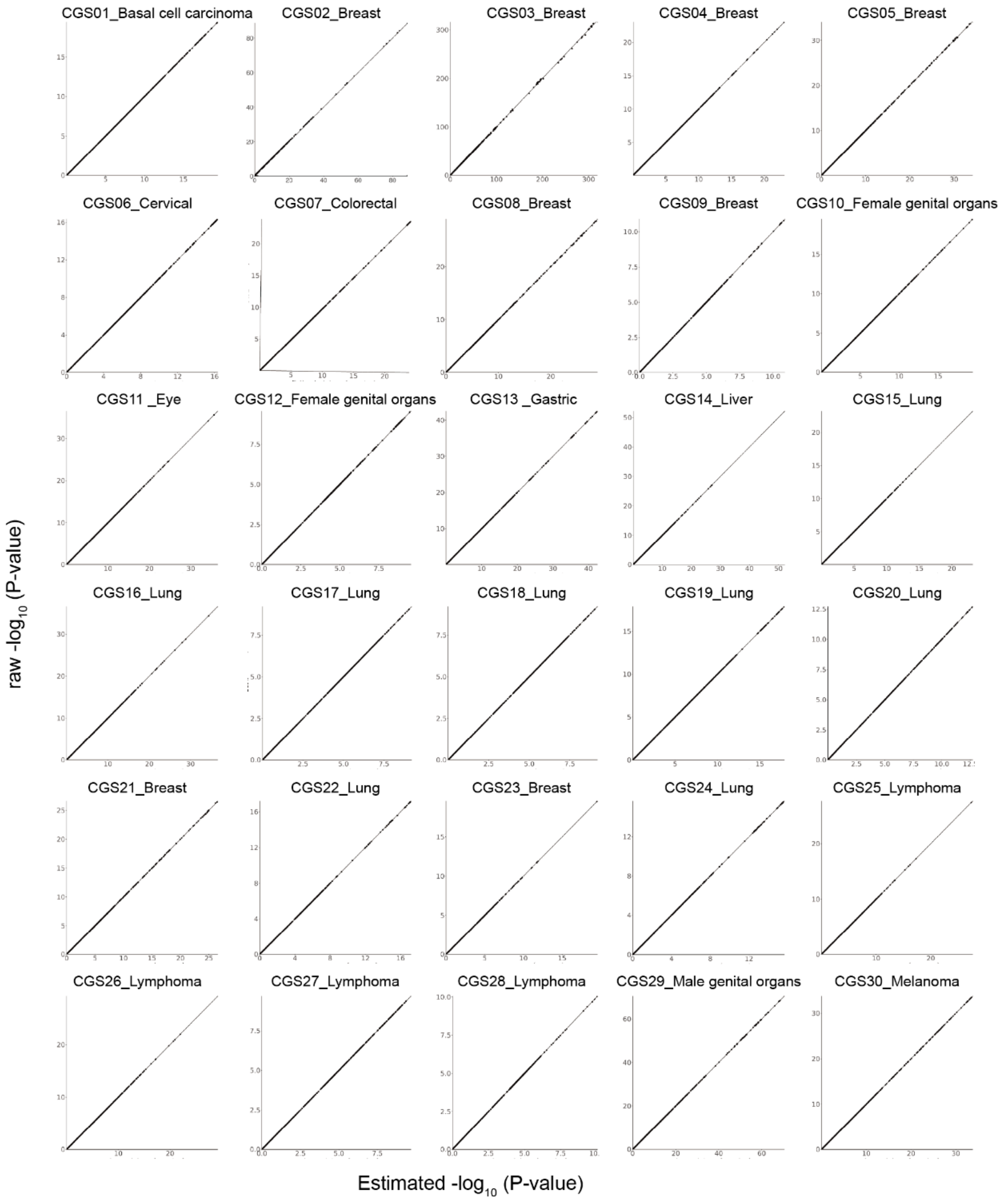
CGS01_Basal cell carcinoma
CGS02_Breast
CGS03_Breast
CGS04_Breast
CGS05_Breast
CGS06_Cervical
CGS07_Colorectal
CGS08_Breast
CGS09_Breast
CGS10_Female genital organs
CGS11 _Eye
CGS12_Female genital organs
CGS13 _Gastric
CGS14_Liver
CGS15_Lung
CGS16_Lung
CGS17_Lung
CGS18_Lung
CGS19_Lung
CGS20_Lung
CGS21_Breast
CGS22_Lung
CGS23_Breast
CGS24_Lung
CGS25_Lymphoma
CGS26_Lymphoma
CGS27_Lymphoma
CGS28_Lymphoma
CGS29_Male genital organs
CGS30_Melanoma
raw -log10 (P-value)
Estimated -log10 (P-value)

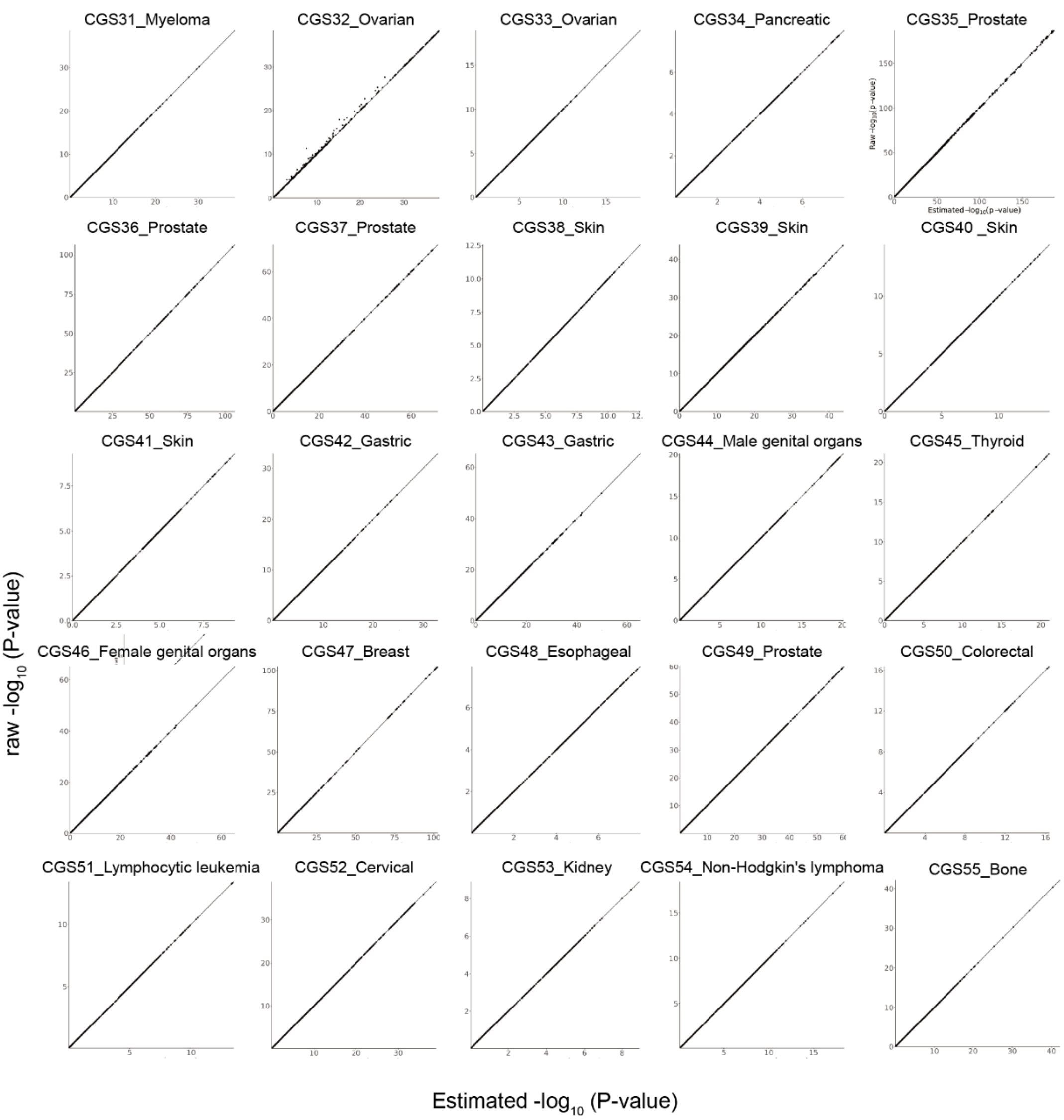

Figure S3. P-Z plot of GWAS summary quality. P-Z -plot shows the quality control for each cancer trait. For the P-Z plot, the X-axis is the Estimated -$\log_{10}$(*P*-value) and the y-axis is the Raw -$\log_{10}$(*P*-value).

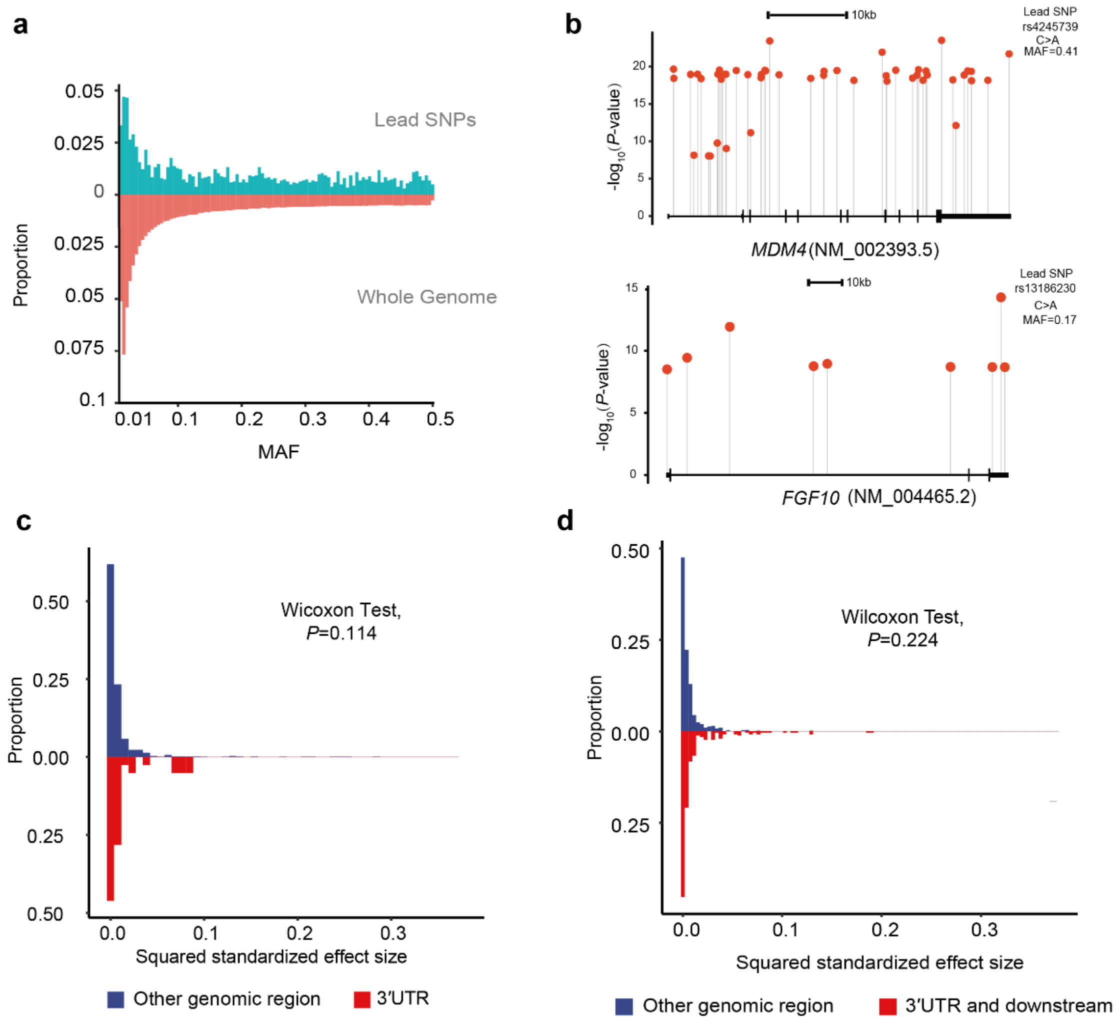

Figure S4. Characterization of cancer GWAS loci. a. Histogram of minor allele frequencies (MAFs) for unique leading SNPs compared with the whole genome. **b.** Examples of genes with lead cancer risk variants were located in 3′UTR regions. **c.** Comparison of the standardized effect size of lead SNPs within 3′UTR, relative to other regions that did not show significant differences. **d.** Comparison of the standardized effect size of lead SNPs within 3′UTR and its downstream relative to other regions. The two-sided *P*-values were obtained using a Wilcoxon sum-rank test.

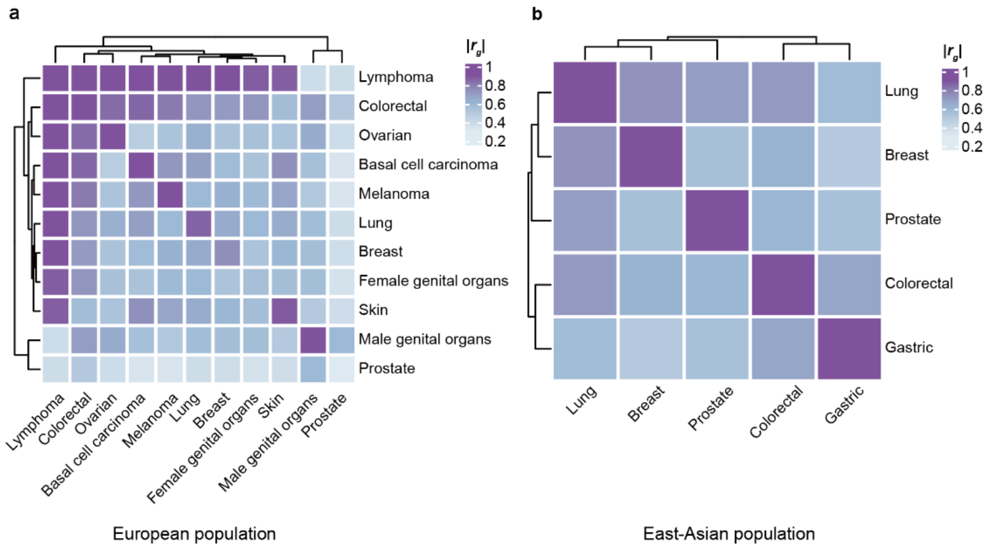

Figure S5. Cross cancer genetic correlation analysis. Heatmaps display the absolute value of the genetic correlation $(|r_g|)$ between different cancer types within **(a)** European and **(b)** East-Asian population. The color in the heatmap represents the correlation between the compared groups, with darker colors indicating stronger correlations.

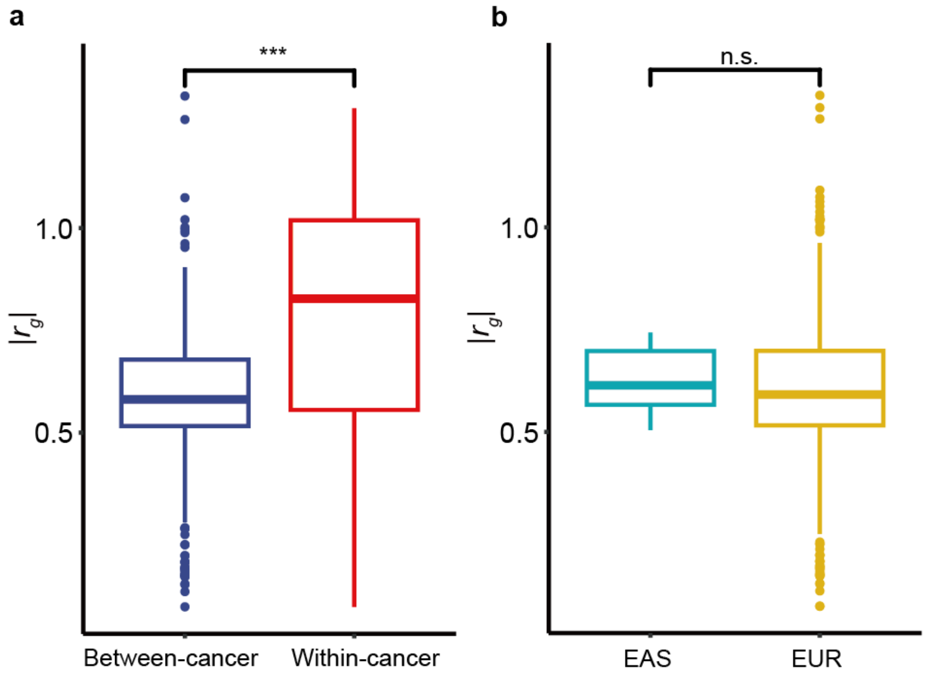

Figure S6. Genetic correlation between different groups. Comparisons of the genetic correlations **a.** within cancers *vs*. between cancers and **b.** in Europeans *vs*. Asians. *P*-values were calculated from the Wilcoxon test (two-sided). **, *P* < 0.01; ****P* < 0.001; ****, *P*<0.0001; ns, not significant. The center lines within the box plot signify the median values, while the boxes encompass the interquartile range (IQR) from the 25th to the 75th percentile and the outliers are shown as separate dots.

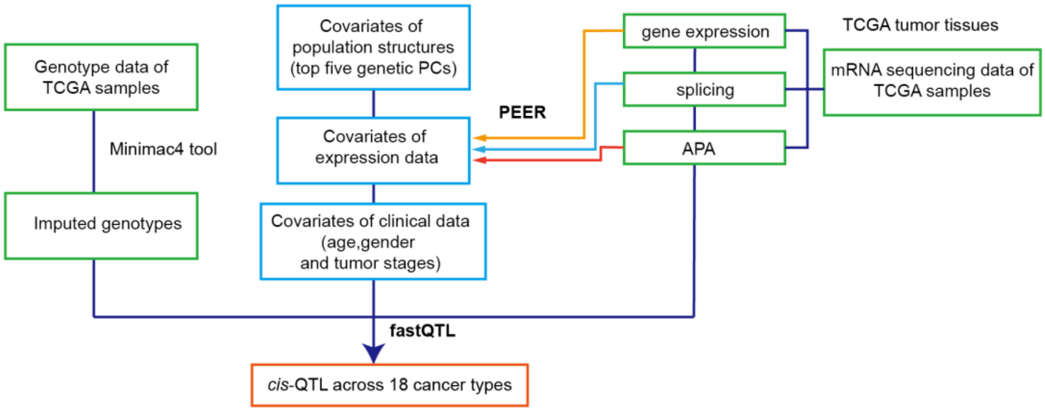

Figure S7. Overview of TCGA xQTL mapping. In this study, we utilized a linear regression framework implemented in Matrix eQTL to assess the association between normalized PDUI values and SNPs within a 1 Mb interval from the 3′UTR region. We adjusted for known covariates, including sex, RIN (RNA Integrity Number), platform, and the top-five genotype principal components. Additionally, we accounted for unobserved covariates using PEER, with the number of PEER covariates determined based on the GTEx Consortium's guidelines. To obtain reliable statistical significance, we conducted 1,000 rounds of permutation and derived empirical *P*-values for each gene. Finally, we applied the R package qvalue to adjust the empirical *P*-values for multiple testing.

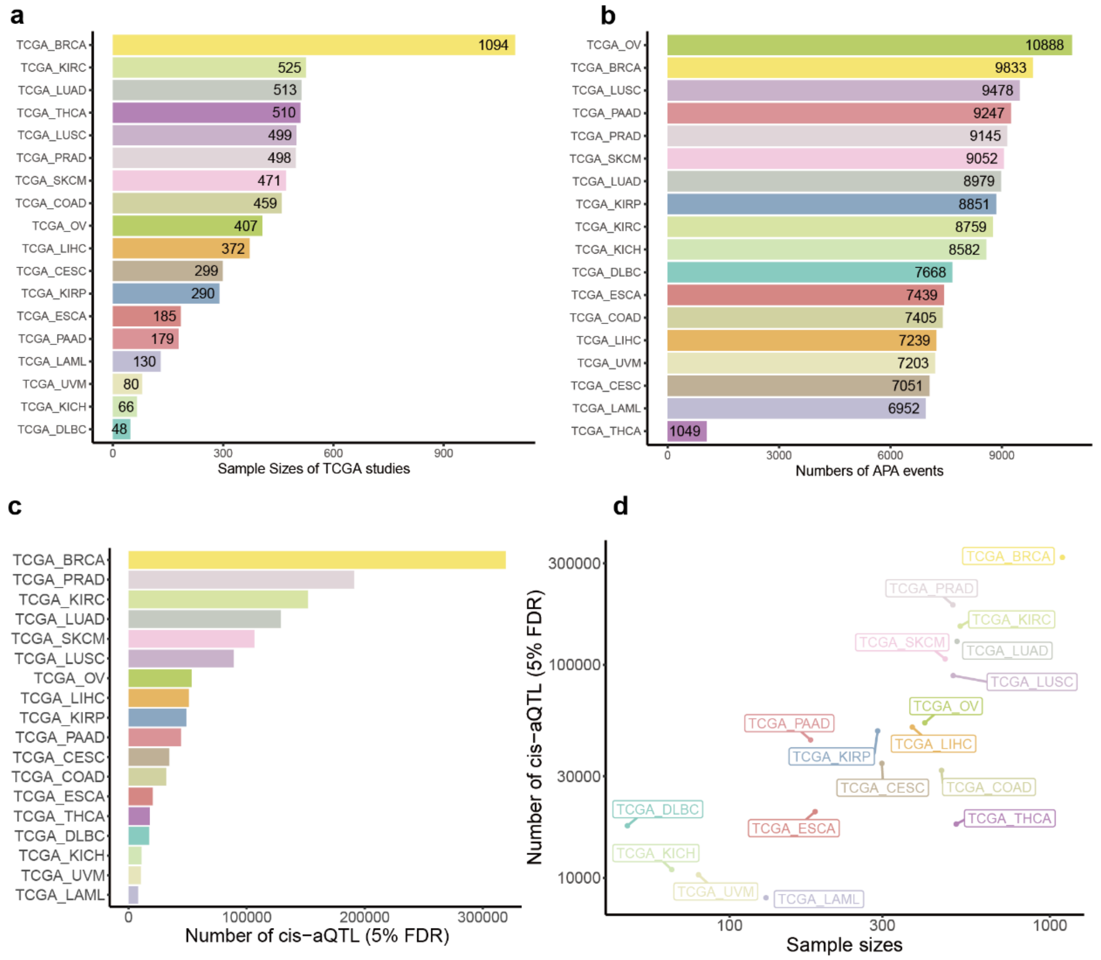

Figure S8. Summary of TCGA 3'aQTLs. a. Sample sizes of TCGA studies included. **b.** Number of APA events detected across different TCGA studies. **c.** Number of cis-3′aQTL identified for each TCGA study (5% FDR). **d.** Correlation between sample size and number of cis-3′aQTL (5% FDR).

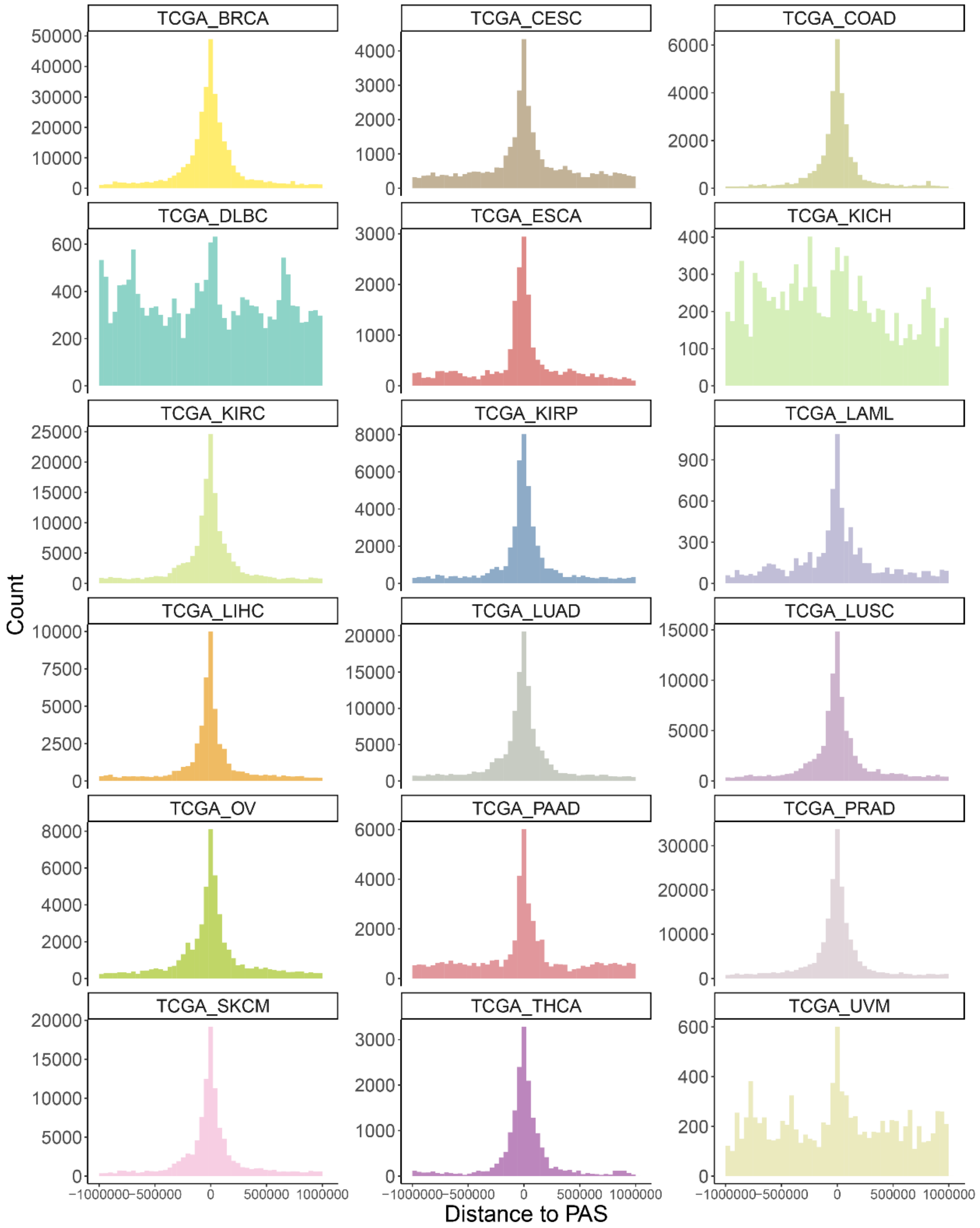

Figure S9. Enrichment of the *cis*-3′aQTL around the PAS. Distance from the PAS to the corresponding SNP in a range of ± 1Mbp. 0 represents PAS.

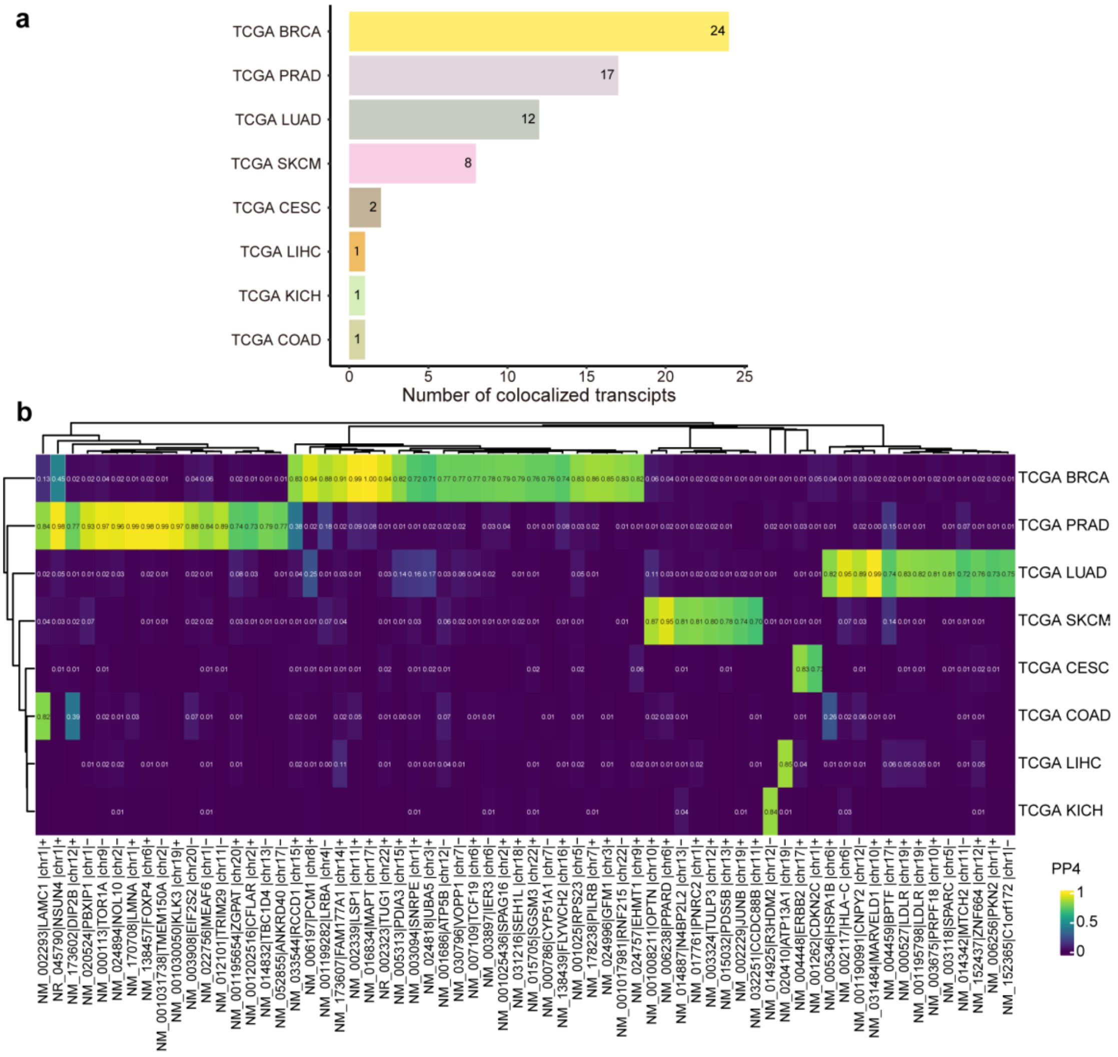

Figure S10. 3′aQTL Colocalization results in TCGA. a. Number of co-localized transcripts per loci. **b.** PPH_4 of co-localization for colocalized genes (PP4>0.7).

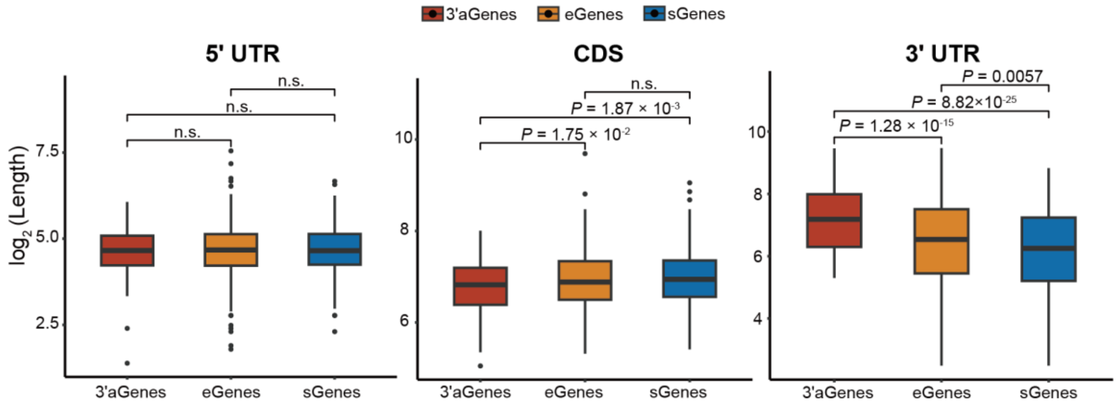

Figure S11. Sequence structure comparisons between colocalized 3′aGenes, eGenes and sGenes. *P* values were determined through a two-sided t-test. The center horizontal lines of the box plot show the median values and the boxes span from the 25th to the 75th percentile. n.s., not significant. The center lines within the box plot signify the median values, while the boxes encompass the interquartile range (IQR) from the 25th to the 75th percentile and the outliers are shown as separate dots.

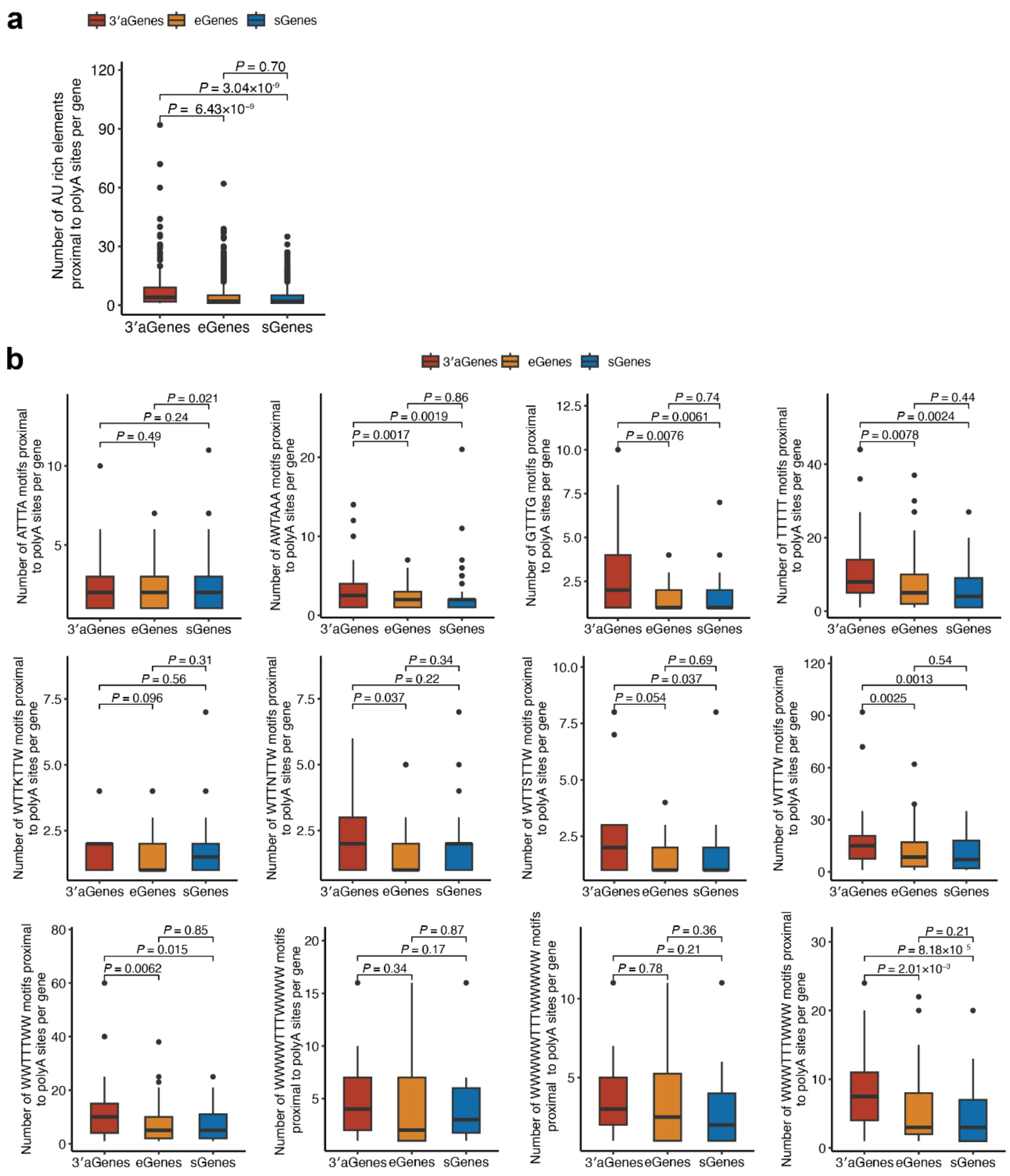

Figure S12. Colocalized 3′aGenes have more AU-rich elements proximal to poly(A) sites compared with eGenes and sGenes. **a.** Boxplot showing the number of overall AU-rich elements proximal to poly(A) sites (ranged from -100 to 100 bp from the poly (A) site) in each gene, comparing 3′aGenes with eGenes and sGenes. Centre horizontal lines of Boxplot represent median values, boxes span from the 25th percentile to the 75th percentile. Whiskers extend to 1.5 × IQR, where IQR is the interquartile range. **b.** Boxplots for each specific motif variant proximal to poly(A) sites in 3′aGenes, eGenes, and sGenes.

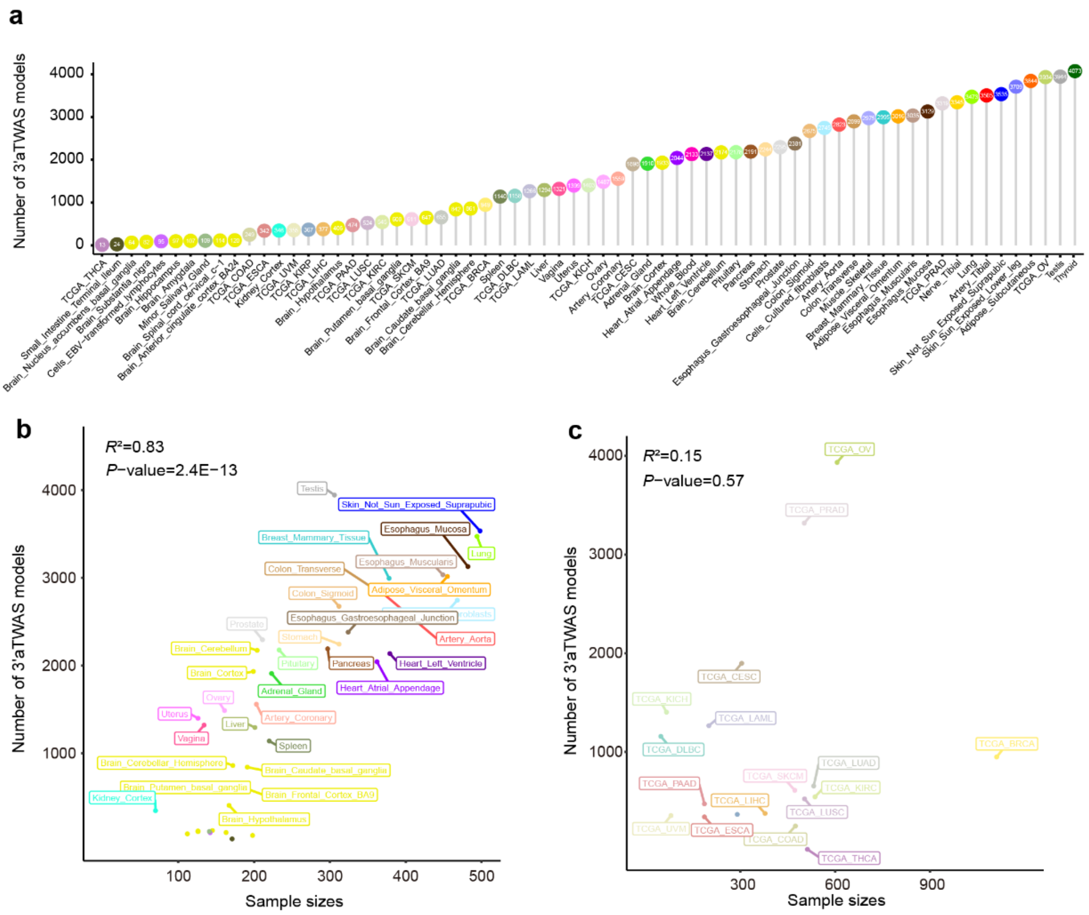

Figure S13. 3′aTWAS models across 49 tissues from GTEx cohorts and 18 tumor tissues from TCGA studies. a. Number of 3'aTWAS models across each tissue from GTEx and TCGA cohorts. **b.** The number of predictive models is highly correlated with the sample size in GTEx tissues. **c.** The number of predictive models is highly correlated with the sample size in TCGA studies. Each dot represents a tissue type.

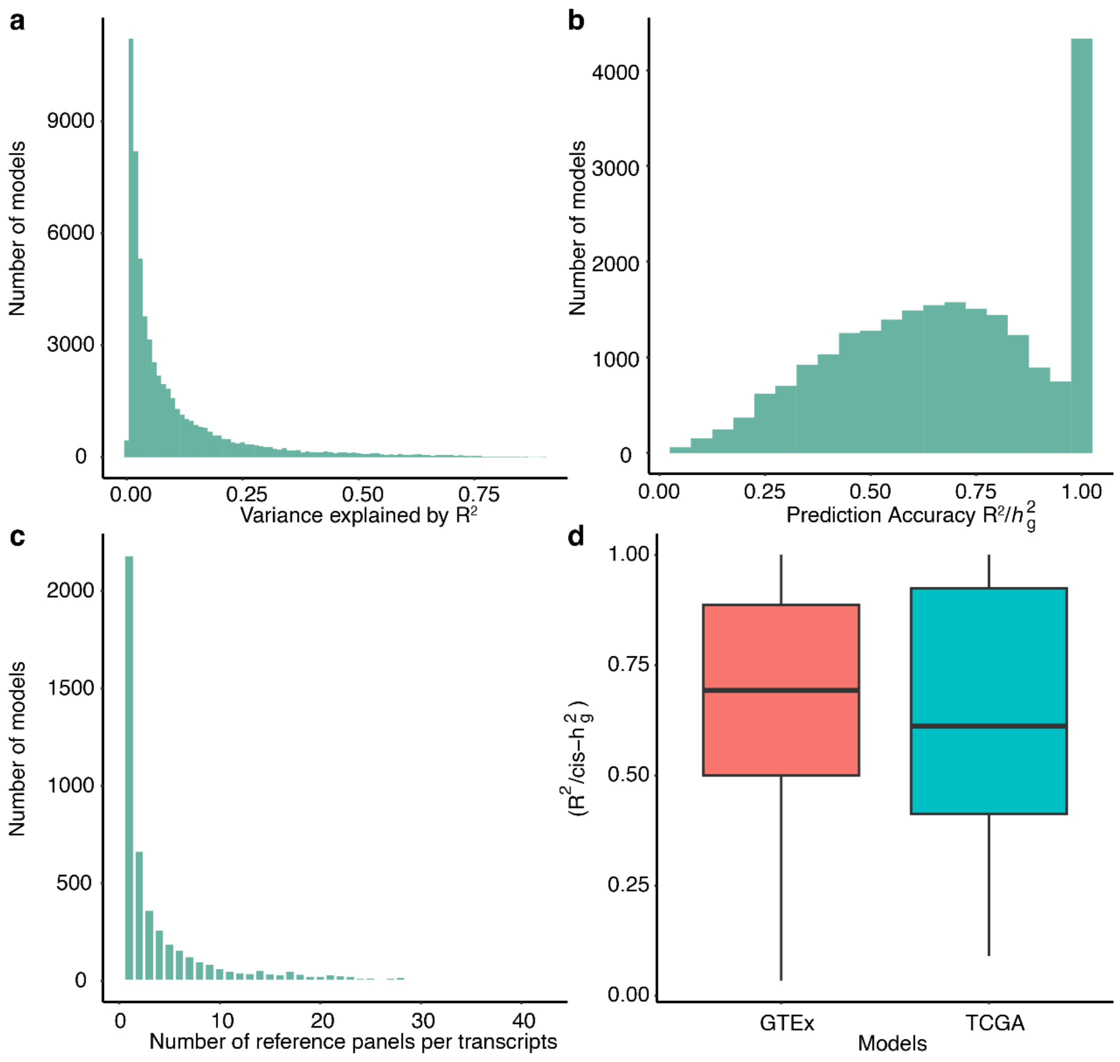

Figure S14. Tissue-specific predictive models for APA usages. a. Cross-validation prediction accuracy of *cis*-regulated APA usage (R^2) for all 64,513 models. **b.** Prediction accuracy ($R^2/cis - h_g^2$) for all 64,513 models, calculated using cross-validation R2 between predicted and true PDUI and normalizing by corresponding cis-h_g^2. **c.** Histogram of the number of reference panels per transcript. The x-axis represents the number of overlapping reference panels for each model, and the y-axis represents the number of 3′aTWAS models. **d.** Boxplot of prediction accuracy for 3′aTWAS models. The median values are represented by the center horizontal line, and the boxes indicate the 25th and 75th percentiles. The median value of the prediction accuracy is 0.69.

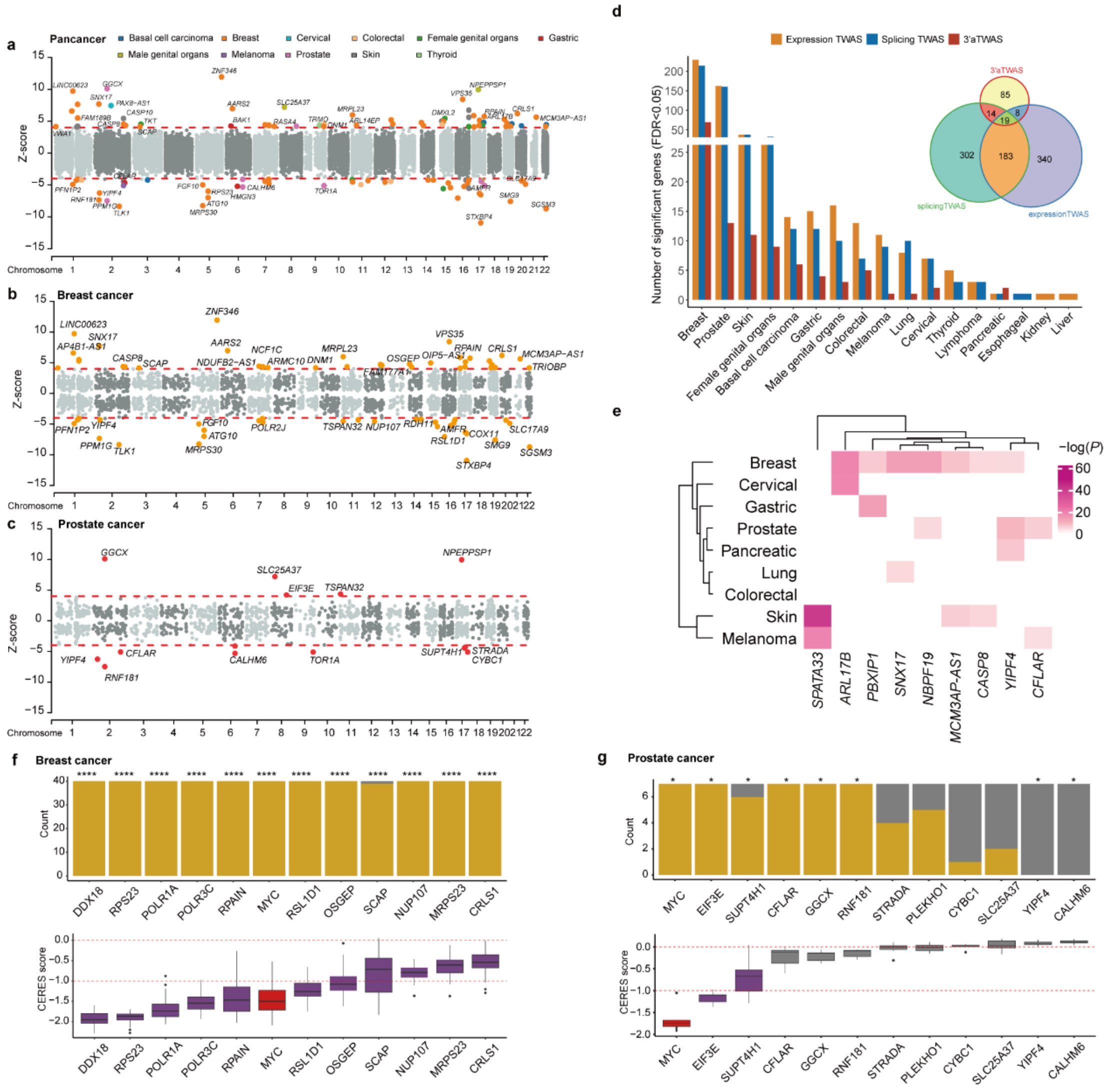

Figure S15. APA Transcriptome-wide association study results based on potentially relevant tissues. a-c. Manhattan plot of 3′aTWAS nominating the APA-linked susceptibility genes in pan-cancer **(a)**, breast cancer **(b),** and prostate cancer **(c)** respectively. Colored points represent significant 3′aTWAS associations at FDR<0.05. **d.** Bar plots showing the number of significant genes detected by 3′aTWAS, with a false-discovery rate (FDR) < 0.05, for different cancers in the most relevant tissues. Venn plot shows the intersection of significant cancer genes identified by 3′aTWAS (FDR <0.05) with genes identified by expression and splicing TWAS. **e.** Heatmap showing the 3′aTWAS genes shared across different cancer types. The color represents the P-values for 3′aTWAS results. **f-g**. Effect of cancer-susceptibility-associated APA-linked genes on cell proliferation of (e) breast cancer (*n* = 45) and (f) prostate cancer-related (n=8) cell lines for potentially relevant tissue. MYC is a known

essential gene set as positive control based on experimental data from DepMap. CERES score to represent the gene essential levels, which corrects for the computational effects of copy number and depletion of gene-targeting guide RNAs. Red dashes denote the median CERES cutoff value of < –0.5, which indicates an essential role in cell proliferation. The significance of cell proliferation was tested for each gene based on the count of CERES values < –0.5 in a total of respective relevant cells using the Binomial test. **, *P*-value < 0.01; *** *P*-value < 0.001; ****, *P*-value <0.0001; ns, not significant. The center lines within the box plot (of Figures S15 f-g) signify the median values, while the boxes in each plot represent the first and third quartiles; the whiskers extend to 1.5 times the IQR and the outliers are shown as separate dots.

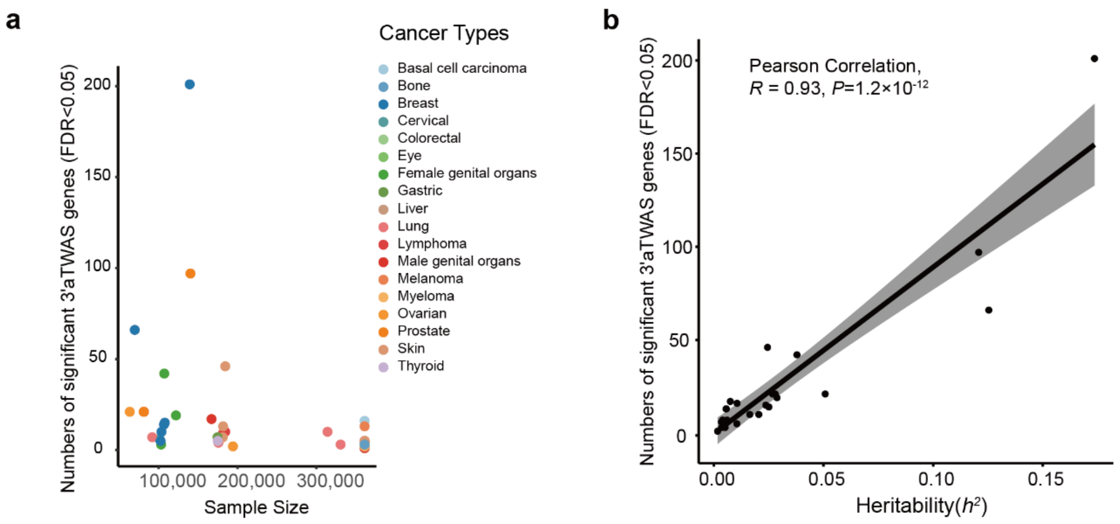

Figure S16. The correlations between sample sizes, heritability, and the number of identified APA-linked genes across cancer types. a. The GWAS sample size shows no significant correlation with the number of identified significant 3'aTWAS genes (FDR<0.05). **b.** There is a strong correlation between estimated heritability and the number of significant 3'aTWAS genes (FDR<0.05). The coefficient (*R*) and *P*-value were calculated from the Pearson correlation.

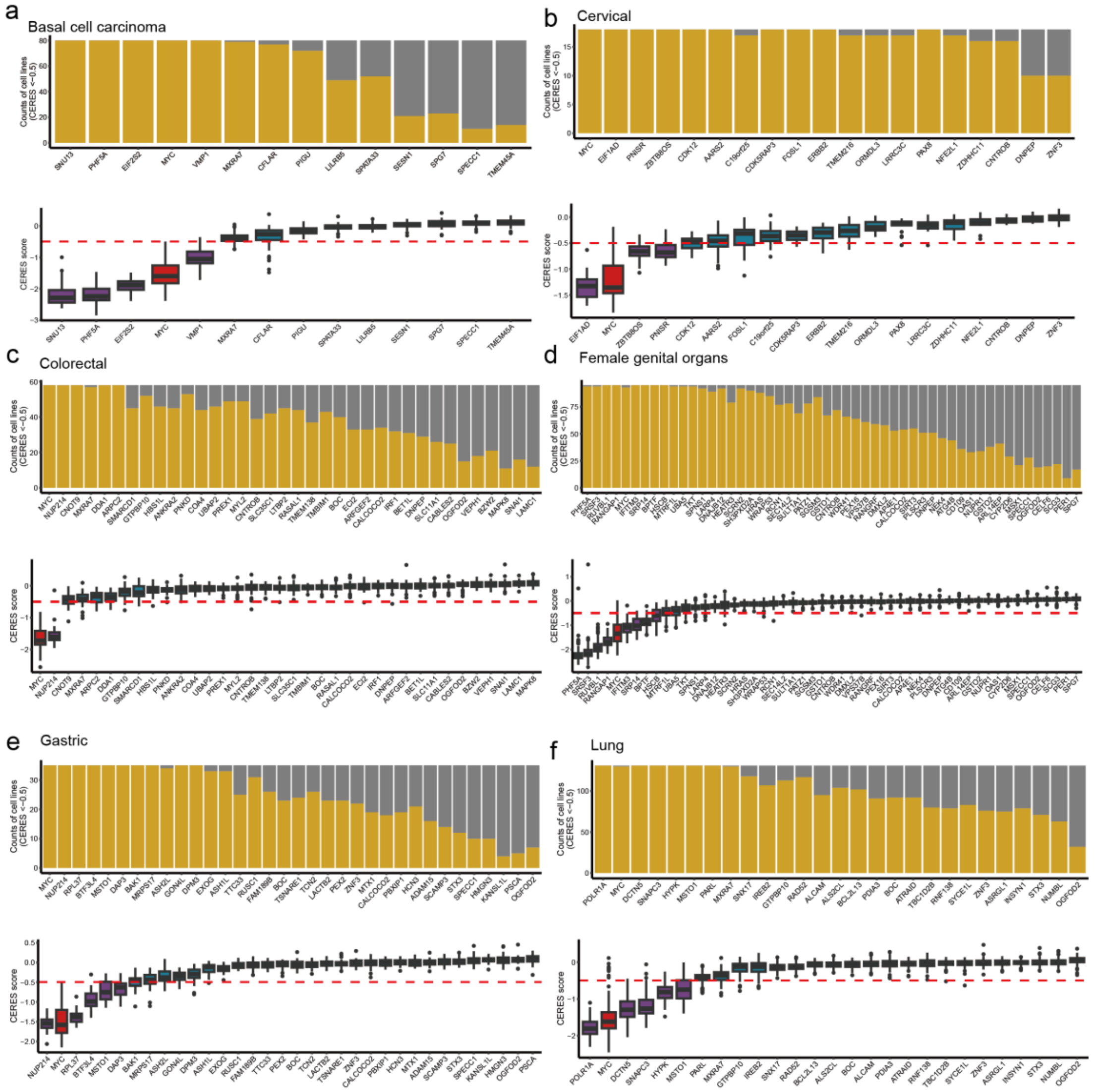
a
Basal cell carcinoma
Counts of cell lines (CERES <-0.5)
CERES score
b
Cervical
c
Colorectal
d
Female genital organs
e
Gastric
f
Lung

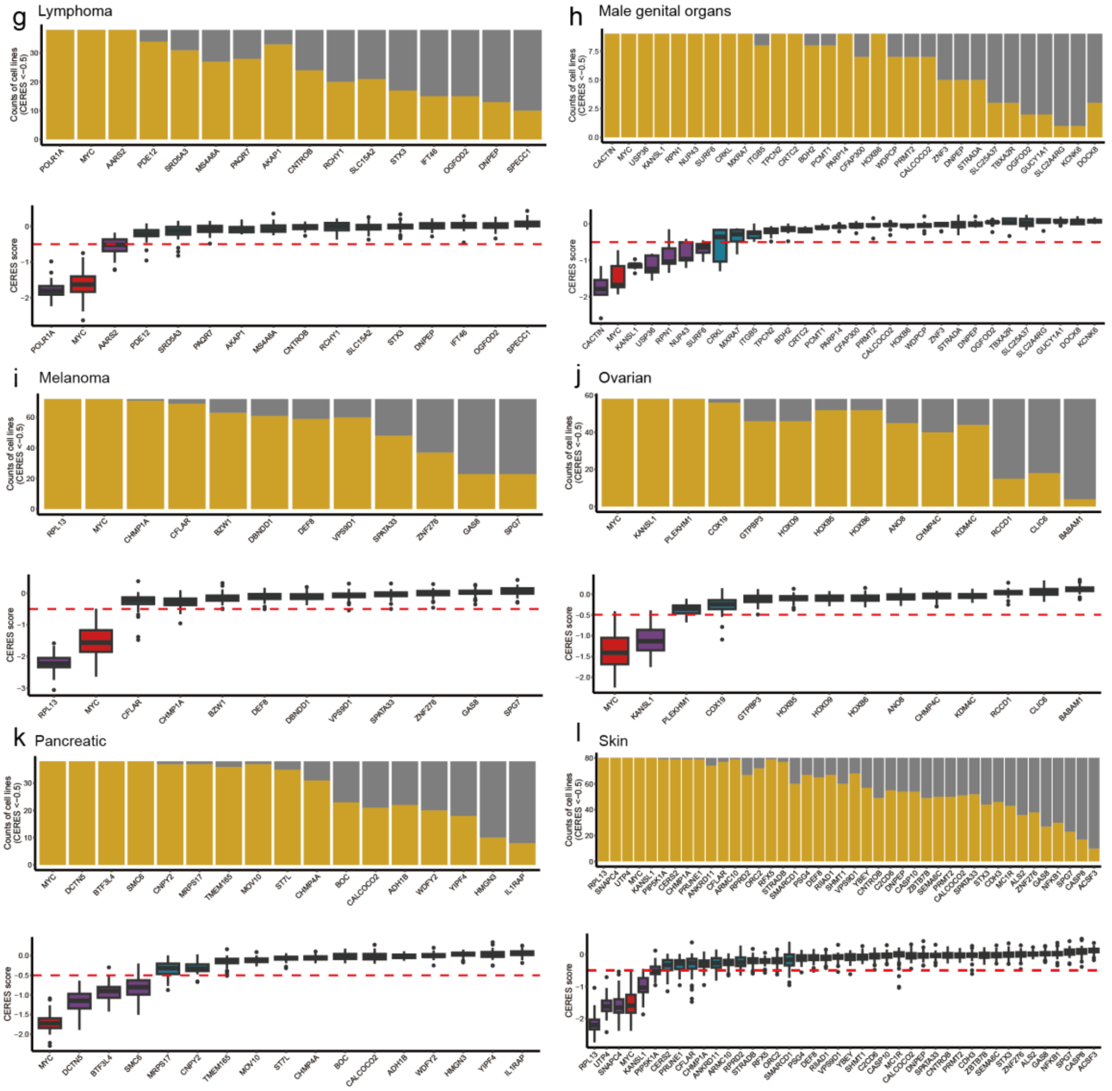

Figure S17. Functional analyses show evidence of essentiality for 3′aTWAS genes in potentially relevant tissues. Gene knockout experiments to determine gene essentiality for different cancer types **(a-l)** in their relevant cell lines. The CERES score indicated a depletion of gene-targeting guide RNAs (corrected by copy number). *MYC* is a known essential gene and was shown as a positive control. Genes with a CERES score ⩽ –0.5 (which is deemed as evidence of essentiality) are with purple in the boxplot. The cutoff of CERES value = –0.5 was shown in a red dashed line. For each gene, we tested its significance on cell proliferation based on the count of negative CERES values in the total number of respective cell lines using the Binomial test. The sample size is shown on the Y-axis of the boxplot.

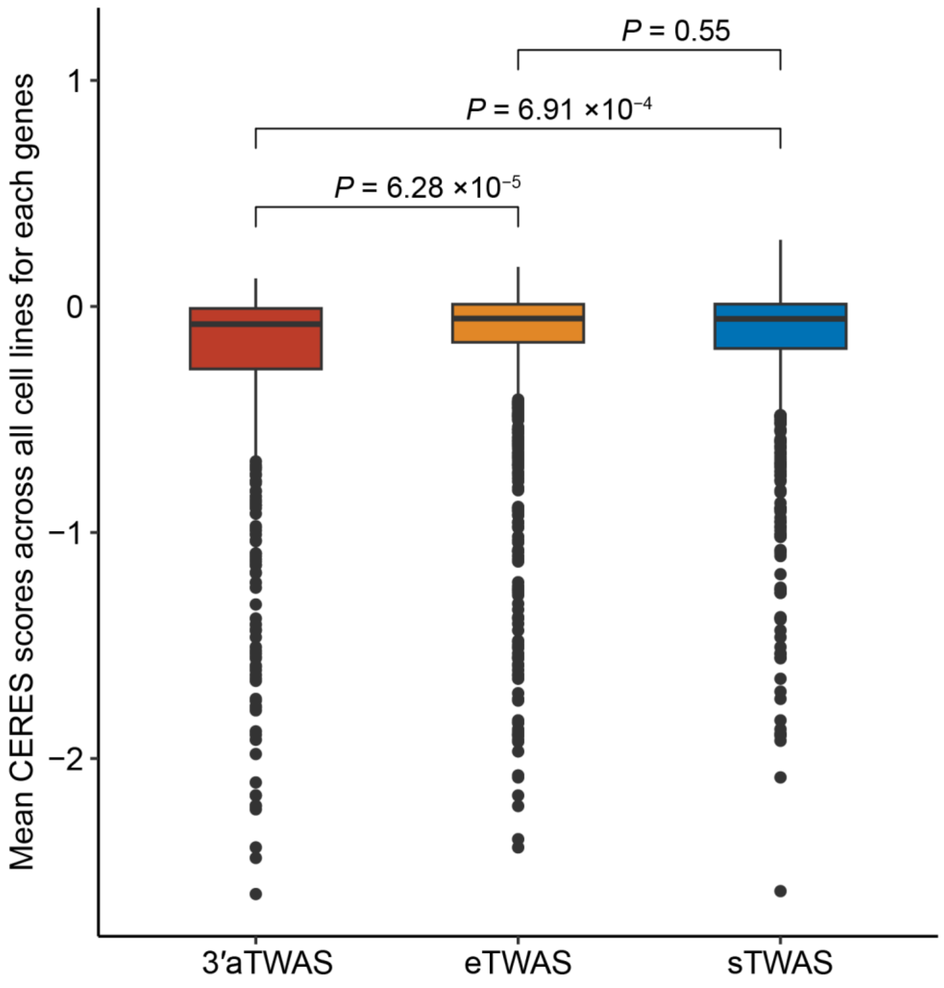

Figure S18. Comparisons of the mean CERES score among significant 3'aTWAS genes versus expression TWAS and splicing TWAS. The *P*-value was calculated from the Wilcoxon's test (two-sided), n=720.

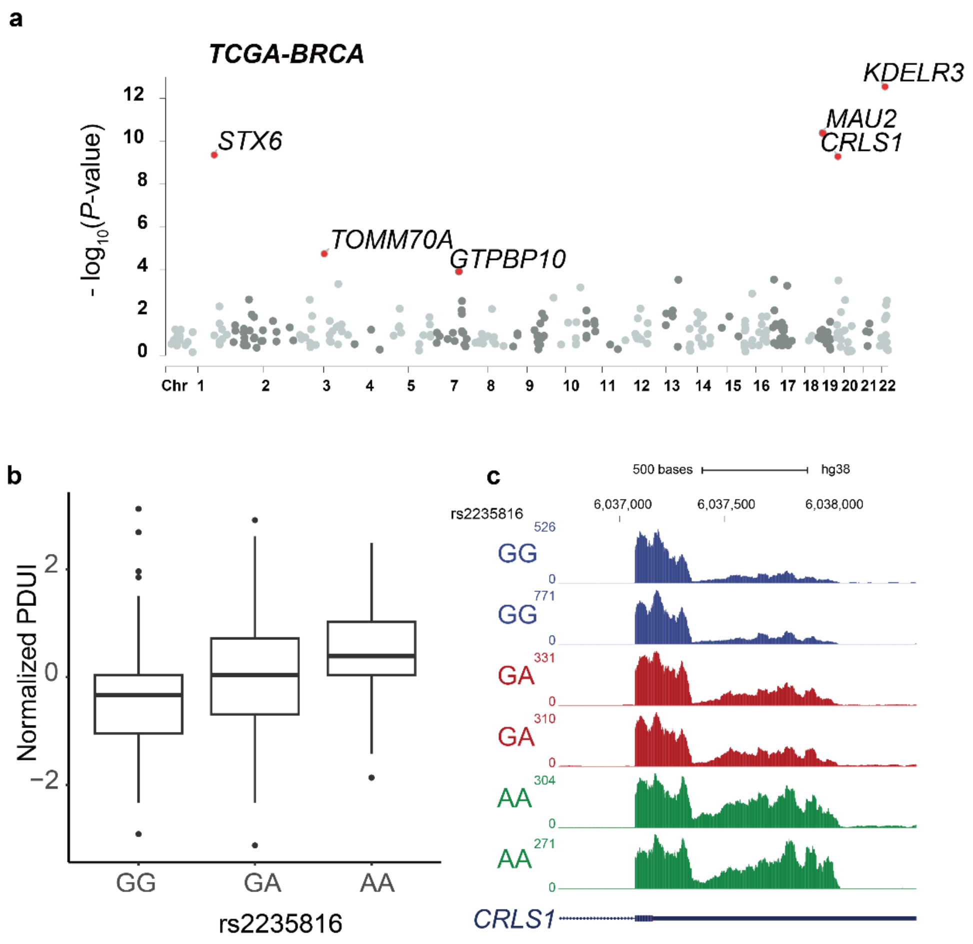

Figure S19. Significant APA-linked susceptibility gene *CRLS1* in TCGA BRCA data. a. Manhattan plot of TWAS results for TCGA-BRCA. **b.** boxplot of rs2235816 for normalized PDUI values in TCGA-BRCA datasets (n = 1,094). The center lines within the box plot signify the median values, while the boxes encompass the interquartile range (IQR) from the 25th to the 75th percentile and the outliers are shown as separate dots. **c.** 3′UTR coverage plot of different alleles of rs2235816 for randomly selected samples in TCGA-BRCA datasets.

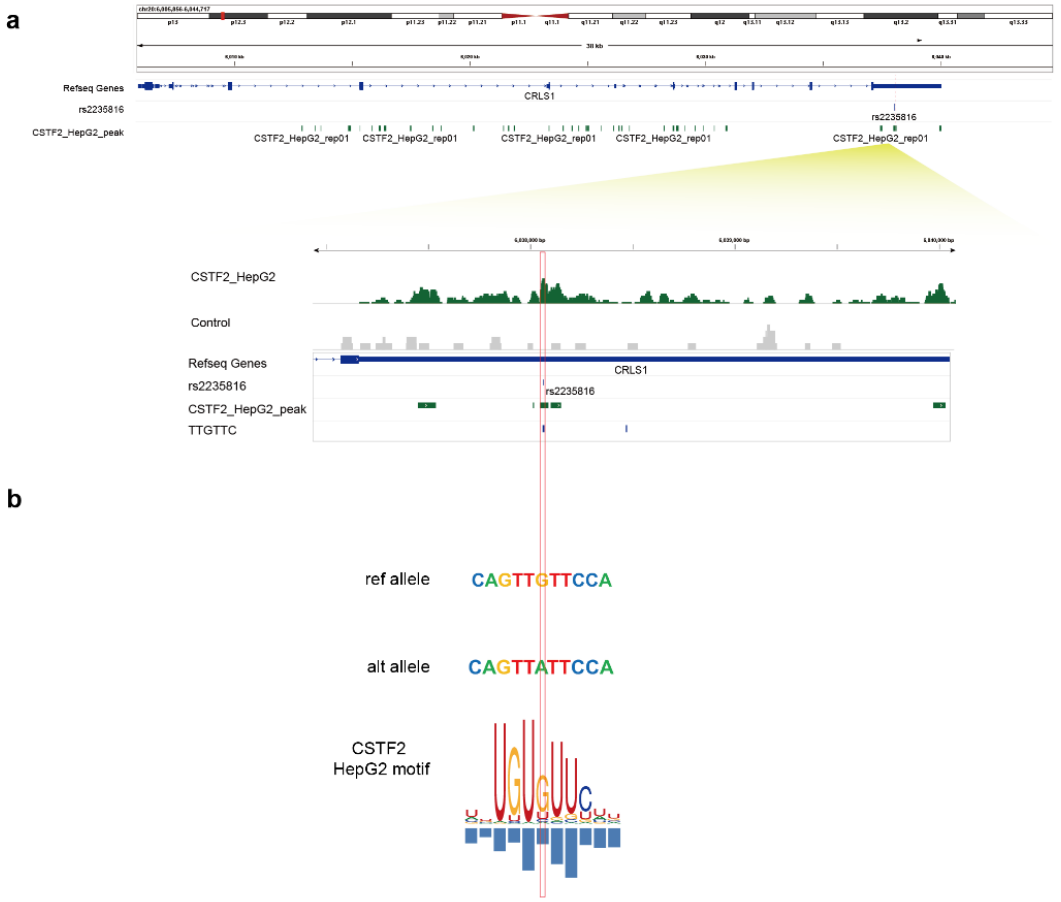

Figure S20. rs2235816 overlaps RNA-binding protein mark of cleavage (CSTF2; region: chr20:6038056-6038077, hg38) in HepG2 cell line. rs2235816 overlaps **(a)** an RNA-binding protein peak for the RNA-binding protein CSTF2 in HepG2 cells and **(b)** a predicted CSTF2 motif.

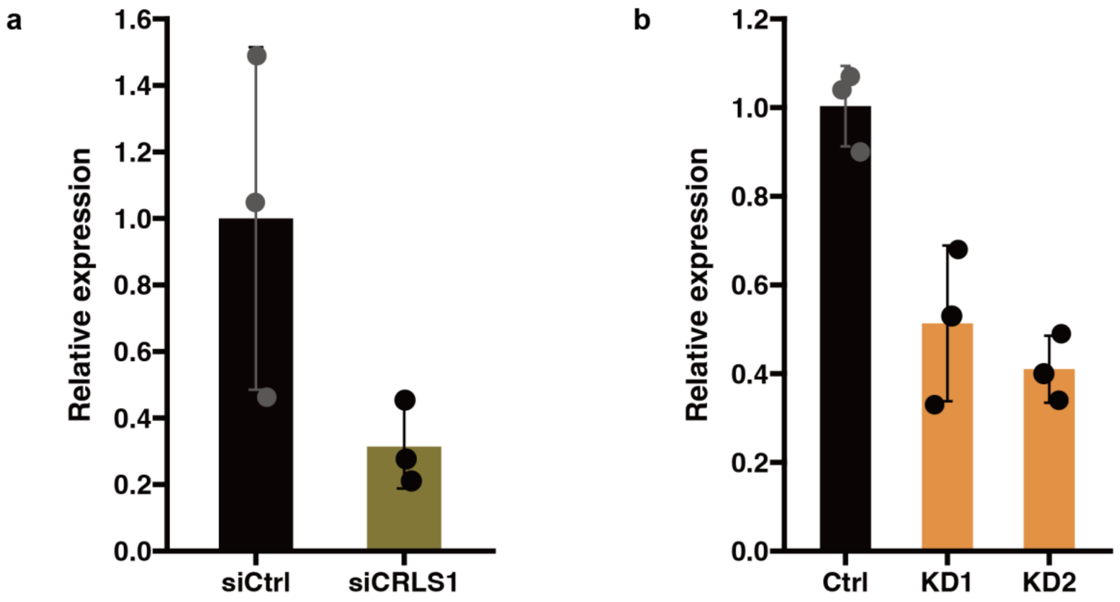

Figure S21. Validation of knockdown efficiency for the indicated siRNAs and shRNAs. Quantitative reverse transcription (rt)-PCR measuring *CRLS1* gene expression of **(a)** the indicated siRNAs and **(b)** shRNAs in MCF-7 cells (*n*=3 independent samples).

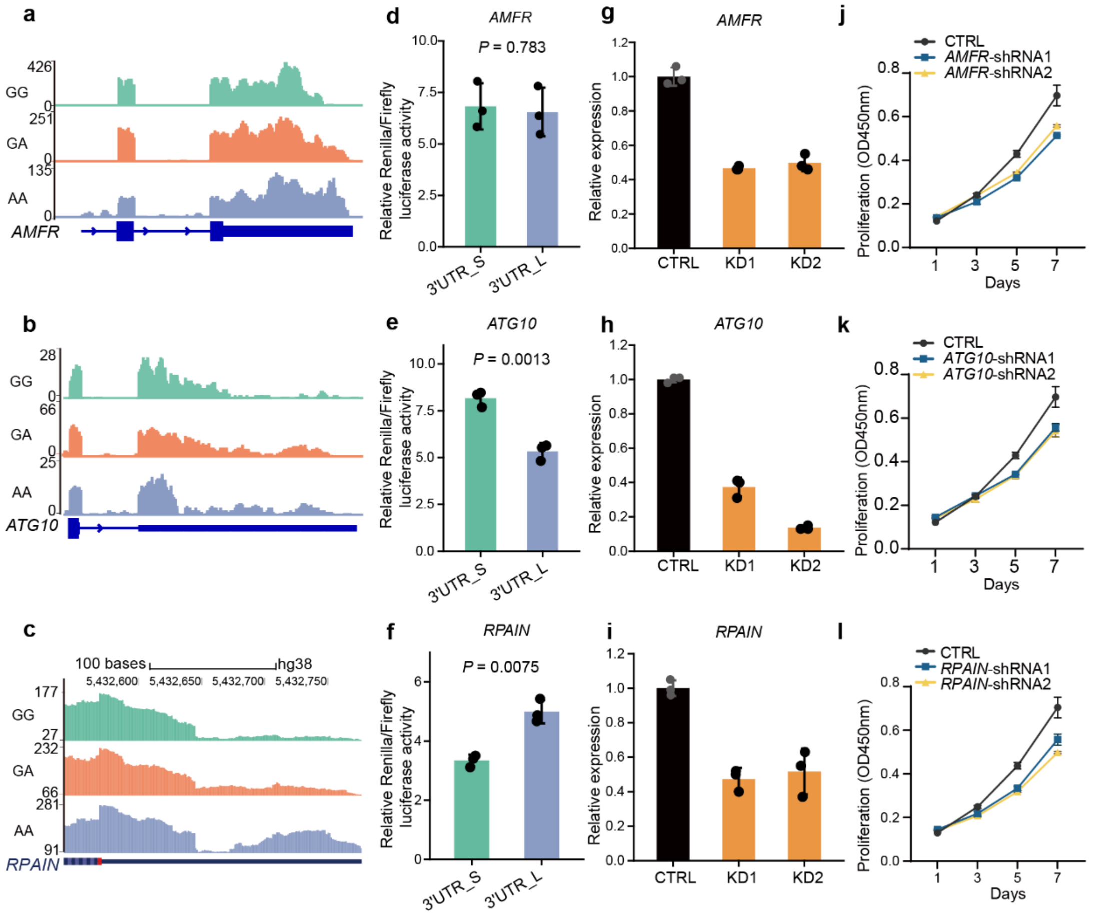

Figure S22. Experimental validation of other breast cancer APA-linked susceptibility genes. a-c. Example of RNA-seq coverage plot for the 3′UTR of indicated genes. **d-f**. Luciferase activity from a reporter system containing the short and long 3′UTR of indicated genes in MCF-7 cells. Significance was determined by the unpaired Student's t-test (two-tailed), comparing the experimental group with the control. *n*=3 independent samples. **g-i.** Quantitative reverse transcription (qRT)-PCR measuring indicated gene expression upon shRNA knockdown in MCF-7 cells. *n*=3 independent samples. **j-l.** Cell proliferation of shRNA-mediated knockdown cells was analyzed on 1, 3, 5, and 7 days. For panels d-f, the bar graphs show the mean ± standard deviation values from three independent experiments.

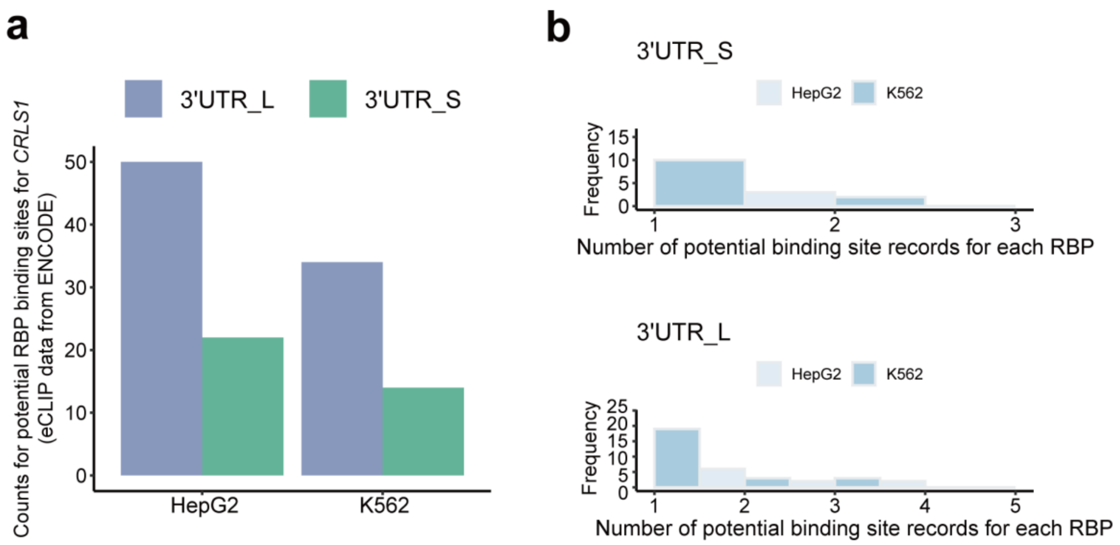

Figure S23. The RBP binding sites for CRLS1 3′UTR region. **a.** Counts for RBP binding sites occurring at *CRLS1*. 3′UTR_L, lengthening 3′UTR region; 3′UTR_S, short 3′ UTR region. **b.** Long 3′ UTR provided more RBP binding sites for each single RBP.

Supplementary Methods

TCGA genotype data imputation and quality control

TCGA germline variants genotype data were obtained from the patient's blood DNA samples and generated using the Affymetrix SNP 6.0 array. Raw CEL format genotype files were obtained from TCGA and using Analysis Power Tools (APT) (https://media.affymetrix.com/support/developer/powertools/changelog/index.html) for quality control and genotype calling. Before imputation, stringent quality control was performed to obtain high quality germline variants data according to these criteria: 1) individuals with SNP call rate less than 90% were removed; 2) SNPs with call rate less than 90% were removed; 3) SNPs with MAF less than 1% were removed; 4) ambiguous SNPs and reversed SNPs, detected using snpflip, were either removed or flipped, respectively. After QC, autochromes SNPs were phased using Eagle v2.4.1 (https://alkesgroup.broadinstitute.org/Eagle/) and imputed with Minimac4 (https://github.com/genepi/imputationserver). The reference panel comes from 1000 genome project phase3, which can be downloaded from https://genome.sph.umich.edu/wiki/Minimac4#Reference_Panels_for_Download. After imputation, the imputed genotype data were filtered according to the following criterions: 1) SNPs with MAF less than 1% were removed; 2) SNPs with Hardy-Weinberg Equilibrium less than 1e-6 were removed.

Transcriptome data collection and processing

The RNA-seq data profiles were obtained from the TCGA data portal (https://gdc-portal.nci.nih.gov/). LeafCutter [1] was used to quantify the alternative splicing events. Expression data profiles were obtained from the Xena [2] (https://tcga.xenahubs.net). PDUI data profiles were obtained from the TC3A [3] (http://tc3a.org). To minimize the effects of outliers on the regression scores, the expression values for each gene across all samples were transformed into a standard normal based on rank.

References

1. Li, Y.I. *et al.* Annotation-free quantification of RNA splicing using LeafCutter. *Nat Genet* **50**, 151-158 (2018).
2. Goldman, M.J. *et al.* Visualizing and interpreting cancer genomics data via the Xena platform. *Nat Biotechnol* **38**, 675-678 (2020).
3. Feng, X., Li, L., Wagner, E.J. & Li, W. TC3A: The Cancer 3' UTR Atlas. *Nucleic Acids Res* **46**, D1027-D1030 (2018).
