# Supplementary material for: A distinct class of pan-cancer susceptibility genes revealed by an alternative polyadenylation transcriptome-wide association study
Source: Nat Commun. 2024 Feb 26;15:1729. doi: 10.1038/s41467-024-46064-7 (PMC10897204; doi:10.1038/s41467-024-46064-7)
Supplement: Supplementary file 3 — Description of Additional Supplementary Files [file 41467_2024_46064_MOESM3_ESM.pdf]

Description of Additional Supplementary Files

File name: Supplementary Data 1
Description: List of cancer GWAS summary statistics and its matched GTEx tissues and TCGA studies. Details of the cancer GWAS summary statistics information.

File name: Supplementary Data 2
Description: Heritability estimates (h^2) for each cancer.
List of the heritability estimates for each cancer GWAS calculated through LDSC.

File name: Supplementary Data 3
Description: 3′aQTL enrichment for cancer GWAS samples.
List of 3′aQTL enrichment for cancer GWAS samples using *fgwas*.

File name: Supplementary Data 4
Description: Significant 3′aQTLs that were colocalized with cancer GWAS risk loci.
List of the genes for which the associated 3′aQTLs co-localized with GWAS signals. PP0 indicates the null model of no association. PP1 and PP2 indicate the probability that causal variants are either associated with disease signals only or with 3′aQTL only, respectively. PP3 indicates the probability that the genetic effects of disease signals and 3′aQTLs are independent. PP4 indicates the probability that disease signals and 3′aQTLs share causal SNPs.

File name: Supplementary Data 5
Description: APA-linked susceptibility genes identified by 3′alternative polyadenylation (APA) transcriptome-wide association study (3′aTWAS).
List of significant (FDR<0.05) APA-linked susceptibility genes identified by 3′alternative polyadenylation (APA) transcriptome-wide association study (3′aTWAS).

File name: Supplementary Data 6
Description: Genes that identified both in colocalization and 3'aTWAS analyses

File name: Supplementary Data 7
Description: Genes that identified both in GTEx and TCGA for 3'aTWAS analyses

File name: Supplementary Data 8
Description: The summary for CERES scores of 3′aTWAS genes showed comparable levels of essentiality.

File name: Supplementary Data 9
Description: List of DNA oligos used in this study.
List of (1) the primers used for 3′RACE and RT-qPCR; (2) DNA oligo sequences for siRNA and shRNA.
