# Supplementary material for: A distinct class of pan-cancer susceptibility genes revealed by an alternative polyadenylation transcriptome-wide association study
Source: Nat Commun. 2024 Feb 26;15:1729. doi: 10.1038/s41467-024-46064-7 (PMC10897204; doi:10.1038/s41467-024-46064-7)
Supplement: Supplementary file 5 — Reporting Summary [file 41467_2024_46064_MOESM5_ESM.pdf]

nature portfolio

Corresponding author(s): Lei LI, LIn Deng and Wei LI

Last updated by author(s): Jan 24,2024

Reporting Summary

Nature Portfolio wishes to improve the reproducibility of the work that we publish. This form provides structure for consistency and transparency in reporting. For further information on Nature Portfolio policies, see our Editorial Policies and the Editorial Policy Checklist.

Statistics

For all statistical analyses, confirm that the following items are present in the figure legend, table legend, main text, or Methods section.

n/a	Confirmed	
☐	☒	The exact sample size (*n*) for each experimental group/condition, given as a discrete number and unit of measurement
☐	☒	A statement on whether measurements were taken from distinct samples or whether the same sample was measured repeatedly
☐	☒	The statistical test(s) used AND whether they are one- or two-sided<br>*Only common tests should be described solely by name; describe more complex techniques in the Methods section.*
☐	☒	A description of all covariates tested
☐	☒	A description of any assumptions or corrections, such as tests of normality and adjustment for multiple comparisons
☐	☒	A full description of the statistical parameters including central tendency (e.g. means) or other basic estimates (e.g. regression coefficient) AND variation (e.g. standard deviation) or associated estimates of uncertainty (e.g. confidence intervals)
☐	☒	For null hypothesis testing, the test statistic (e.g. *F*, *t*, *r*) with confidence intervals, effect sizes, degrees of freedom and *P* value noted<br>*Give P values as exact values whenever suitable.*
☐	☒	For Bayesian analysis, information on the choice of priors and Markov chain Monte Carlo settings
☒	☐	For hierarchical and complex designs, identification of the appropriate level for tests and full reporting of outcomes
☐	☒	Estimates of effect sizes (e.g. Cohen's *d*, Pearson's *r*), indicating how they were calculated

Our web collection on statistics for biologists contains articles on many of the points above.

Software and code

Policy information about availability of computer code

Data collection	Dual-luciferase assay: Biotek Gen5 3.08, RT-qPCR: Biorad CFX Manager 3.1
Data analysis	STAR v2.5.2b, PEER v1.3, GCTA v1.93, bcftools v1.11, Matrix eQTL v2.1.0, Coloc v5.1-1, R 3.6.2, Image lab 6.1. The open-source DaPars v2 program is freely available at https://github.com/3UTR/DaPars2, ANNOVAR(https://annovar.openbioinformatics.org/en/latest/),CAUSALdb(https://github.com/mulinlab/CAUSALdb-finemapping-pip),CAVIAR(https://github.com/fhormoz/caviar),LD score regression (https://github.com/bulik/ldsc),fgwas(https://github.com/joepickrell/fgwas),Plink 1.90 (https://github.com/bulik/ldsc), PrediXcan (https://github.com/hakyim/PrediXcan),FUSION (http://gusevlab.org/projects/fusion/), STRING 11.5(https://string-db.org/),cytoscape 3.9.1(https://cytoscape.org/).The custom source codes to perform the data analysis relevant to this paper are available, under the MIT license, on Zenodo with the access code DOI https://doi.org/10.5281/zenodo.8223680 and Github https://github.com/lilab-bioinfo/CancerAPA.

For manuscripts utilizing custom algorithms or software that are central to the research but not yet described in published literature, software must be made available to editors and reviewers. We strongly encourage code deposition in a community repository (e.g. GitHub). See the Nature Portfolio guidelines for submitting code & software for further information.

Data

Policy information about availability of data

All manuscripts must include a data availability statement. This statement should provide the following information, where applicable:

- Accession codes, unique identifiers, or web links for publicly available datasets
- A description of any restrictions on data availability
- For clinical datasets or third party data, please ensure that the statement adheres to our policy

Raw whole transcriptome and genome sequencing data from the Genotype-Tissue Expression (GTEx) project are available via the database of Genotypes and Phenotypes (dbGaP) under the accession number: phs000424.v8.p2[https://www.ncbi.nlm.nih.gov/projects/gap/cgi-bin/study.cgi?study_id=phs000424.v8.p2]. All processed GTEx data are available via the GTEx portal((http://gtexportal.org/). Publicly RNA-seq and genotype data from The Cancer Genome Atlas (TCGA) from the Genomic Data Commons (GDC) Data Portal (https://portal.gdc.cancer.gov/). Expression data profiles were obtained from the Xena 2 (https://tcga.xenahubs.net). PDUI data profiles were obtained from the TC3A 3 (http://tc3a.org). GWAS summary statistics are from NHGRI-EBI GWAS catalog (https://www.ebi.ac.uk/gwas/), UK Biobank GWAS, http://www.nealelab.is/uk-biobank/), Finn Gen(https://www.finngen.fi/en) and JENGER(http://jenger.riken.jp). The details, including accession numbers, of GWAS summary statistics used in this study, are listed in Supplementary Data 1. 1000 Genomes Project Reference for LDSC, https://data.broadinstitute.org/alkesgroup/LDSCORE/1000G_Phase3_plinkfiles.tgz; 1000 Genomes Project Reference with regression weights for LDSC, https://data.broadinstitute.org/alkesgroup/LDSCORE/1000G_Phase3_weights_hm3_no_MHC.tgz). All significant 3'aTWAS genes in cancer are available in Supplementary Data 5. The expression and splicing TWAS models for GTEx v8 are publicly available at PredictDB (https://predictdb.org/).

Research involving human participants, their data, or biological material

Policy information about studies with human participants or human data. See also policy information about sex, gender (identity/presentation), and sexual orientation and race, ethnicity and racism.

Reporting on sex and gender	Sex and gender information was determinated based on the existing publicly available GWAS and datasets from GTEx v8 and TCGA
Reporting on race, ethnicity, or other socially relevant groupings	The GWAS analyzed in this study consisted of two ancestral backgrounds: European and East Asian. The ethnicity of GTEx and TCGA were fully described in the original publications referenced in our manuscript. The tissues' donors for the GTEx and TCGA datasets analyzed in this paper were primarily of European descendent.
Population characteristics	The characteristics of participants in the datasets we analyzed are provided in the original publications.
Recruitment	We were not involved in the design of the recruitment procedure for the sequencing datasets analyzed.
Ethics oversight	All of the data described in the manuscript is based on existing publicly available data, the Enthics' statement were described in the original publications.

Note that full information on the approval of the study protocol must also be provided in the manuscript.

Field-specific reporting

Please select the one below that is the best fit for your research. If you are not sure, read the appropriate sections before making your selection.

☒ Life sciences ☐ Behavioural & social sciences ☐ Ecological, evolutionary & environmental sciences

For a reference copy of the document with all sections, see nature.com/documents/nr-reporting-summary-flat.pdf

Life sciences study design

All studies must disclose on these points even when the disclosure is negative.

Sample size	Sample size was determined based on the availability of existing GTEx and TCGA data.
Data exclusions	RNA-seq samples without matched WGS data were excluded. When multiple samples were derived from the same individuals,we selected the samples with the highest RNA integrity numbers(RIN).
Replication	The experiments has been performed independently with biological triplicates, and all attempts at replication were successful.
Randomization	The samples have been assigned randomly according to the beginning experiments.
Blinding	The bioinformatic analyses have been corroborated with blinded wet lab experiments.<br>No formal blinding was used for the experiments due to the predetermined nature and protocol of measurements and the work does not involve participant groups.

Reporting for specific materials, systems and methods

We require information from authors about some types of materials, experimental systems and methods used in many studies. Here, indicate whether each material, system or method listed is relevant to your study. If you are not sure if a list item applies to your research, read the appropriate section before selecting a response.

Materials & experimental systems

n/a	Involved in the study
☒	☐ Antibodies
☐	☒ Eukaryotic cell lines
☒	☐ Palaeontology and archaeology
☒	☐ Animals and other organisms
☒	☐ Clinical data
☒	☐ Dual use research of concern
☒	☐ Plants

Methods

n/a	Involved in the study
☒	☐ ChIP-seq
☒	☐ Flow cytometry
☒	☐ MRI-based neuroimaging

Eukaryotic cell lines

Policy information about cell lines and Sex and Gender in Research

Cell line source(s)	MCF7 and 293T cell lines were obtained from cell resource center of Shanghai Institutes for Biological Sciences, Chinese Academy Science, Shanghai, China.
Authentication	AMFR, ATG10, CRLS1,RPAIN knock-down cell lines were authenticated using RT-qPCR. Authentication of 293T cells was not performed.
Mycoplasma contamination	All cell lines are tested negative for mycoplasma contamination
Commonly misidentified lines (See ICLAC register)	No commonly misidentified cell lines were used in this study

Plants

Seed stocks	Not applicable.
Novel plant genotypes	Not applicable.
Authentication	Not applicable.
