# Supplementary figures and images for: A distinct class of pan-cancer susceptibility genes revealed by an alternative polyadenylation transcriptome-wide association study
Source: Nat Commun. 2024 Feb 26;15:1729. doi: 10.1038/s41467-024-46064-7 (PMC10897204; doi:10.1038/s41467-024-46064-7)

Figure 5e

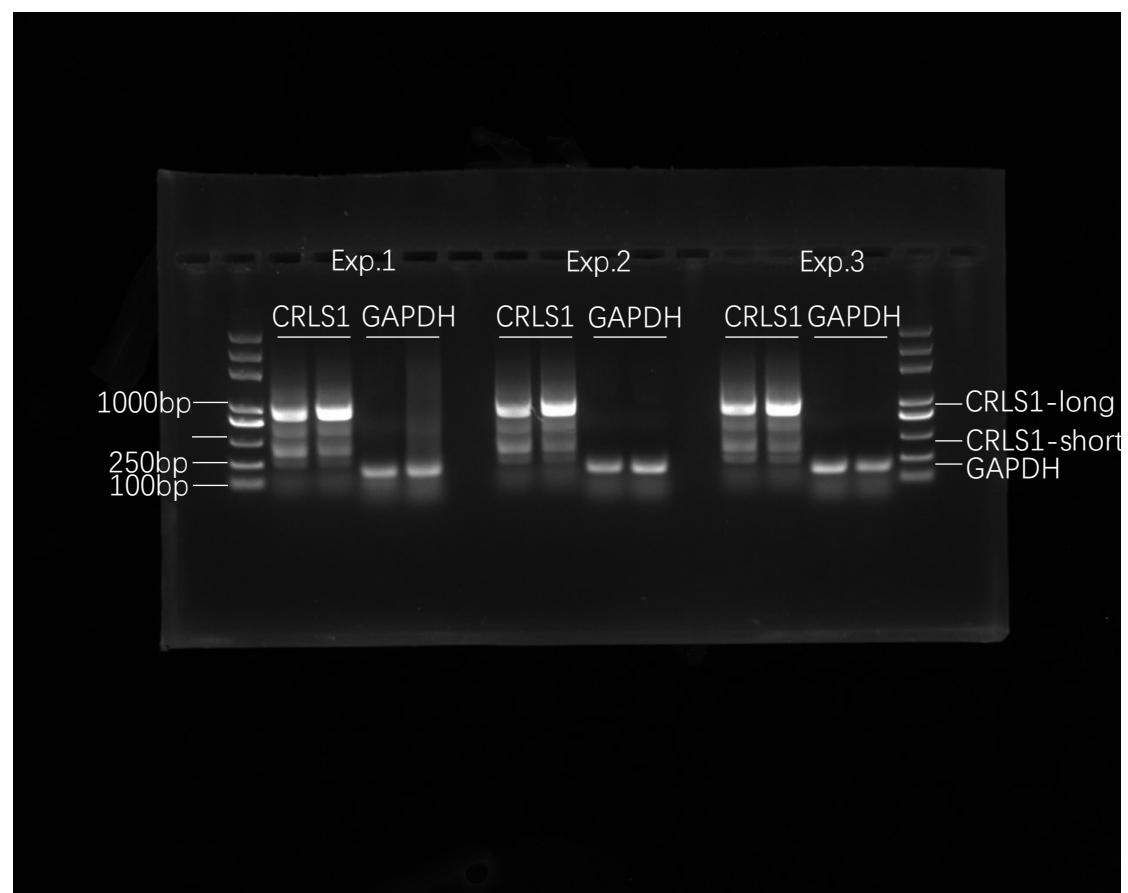

Supplement: Supplementary file 6 — Source Data [file 41467_2024_46064_MOESM6_ESM.zip › Source data.pdf]
